# Premenstrual Syndrome’s Impact on Work-Related Quality of Life Among Jordanian Nurses

**DOI:** 10.7759/cureus.53427

**Published:** 2024-02-01

**Authors:** Yamamah Al-Hmaid, Othman Beni Yonis, Mais Alkhalili, Khalid Kheirallah

**Affiliations:** 1 Department of Public Health and Community Medicine, Faculty of Medicine, Al-Balqa Applied University, Al-Salt, JOR; 2 Department of Public Health and Family Medicine, Faculty of Medicine, Jordan University of Science and Technology, Irbid, JOR; 3 Department of Public Health, Jordan University of Science and Technology, Irbid, JOR

**Keywords:** jordanian nurses, premenstrual symptoms screening tool, work-related quality of life (wrqol) scale, female nurses, premenstrual syndrome

## Abstract

Introduction

Premenstrual syndrome (PMS) is a regular clinical condition that affects most women during their reproductive years. Its related symptoms may be linked to a decrease in women's quality of life. Female nurses may be more susceptible to PMS due to the demanding nature of their job. The importance of nurses’ jobs and how their quality of life will affect patients and themselves in parallel make female nurses a population that is worth investigating. Our objective is to estimate the level of PMS among Jordanian nurses and assess the potential impact of PMS on work-related quality of life among them.

Methods

A cross-sectional study was conducted on 210 nurses who completed a questionnaire regarding demographic data, menstrual characteristics, the Premenstrual Symptoms Screening Tool (PSST), and the Work-Related Quality-of-Life Scale (WRQoL). The nurses were classified as having or not having PMS according to the PSST.

Results

The prevalence of PMS was 60.5%, the results showed a significantly lower mean WRQoL score for nurses with PMS (mean = 65.47, SD = 15.38) compared to nurses without PMS (mean = 70.54, SD = 14.47). The multivariable regression model revealed that the adjusted odds ratios for age, combined oral contraceptive pill (COCP) use, family history, severe dysmenorrhea, job and career satisfaction, and stress at work were 0.90 (95% CI = 0.84, 0.96), 5.18 (95% CI = 1.33, 20.17), 2.52 (95% CI = 1.23, 5.18), 11.78 (95% CI = 2.48, 56.02), 0.92 (95% CI = 0.85, 0.99), and 1.20 (95% CI = 1.01, 1.42), respectively.

Conclusion

PMS is quite prevalent among Jordanian nurses, with a negative impact on their work-related quality of life. Healthcare managers might implement special regulations for female nurses with PMS to improve their work-related quality of life. This research suggests that PMS is a substantial factor in the low work-related quality of life among Jordanian nurses.

## Introduction

Premenstrual syndrome (PMS) is a common clinical condition that occurs in most women during their reproductive years. [1]. The American College of Obstetricians and Gynecologists (ACOG) defines it as "a clinical condition characterized by the cyclic presence of physical and emotional symptoms unrelated to any organic disease that appear during the 5 days before menses in each of the three prior menstrual cycles and disappear within 4 days of the onset of menses without recurrence until at least cycle day 13" [2]. A key aspect of PMS is that the timing and intensity of the symptoms may be severe enough to interfere with regular daily activities. [3].

According to epidemiological studies, up to 80% of women can experience a variety of irritating PMS symptoms [1]. Moreover, there are more than 200 related symptoms of PMS [3] that vary from psychological to behavioral to physical, including headache, mastalgia, bloating, backache, mood changes, anxiety, anger, irritability, lack of interest, change in sleep pattern, change in appetite, and several more symptoms [1,4].

PMS is responsible for causing deterioration in health from physical to psychosocial aspects [4]; it can affect women themselves, their families, and their societies [5]. Furthermore, it has an effect on the nation’s development through high levels of psychological instability and a reduction in women’s daily achievements [6]. In aggregate, PMS can engender challenges spanning approximately 3,000 days throughout a woman's reproductive lifespan [5].

Furthermore, PMS-related somatic and mood symptoms may be associated with a decrease in the quality of life of affected women [7]. PMS can have a negative impact on physical activities and social life, as well as interfere with personal relationships and work performance, lowering the overall quality of life [8]. Many studies have found that moderate-to-severe PMS symptoms can have a negative impact on women's work performance through increased absence and lower productivity, resulting in a decrease in women's quality of life [3]. Previous research defined work-related quality of life for working women as an employee's perception of physical and mental aspects of their working circumstances and factors related to the work environment [9].

Nurses are known to have a high workload and a poor working environment, with regular night shifts [10]. The environment also offers inadequate physical security due to regular contact with people’s diseases and needs and sustained interaction with colleagues and patients’ families [10]. Furthermore, their clinical duties are often critical. Hard and demanding work, occupational hazards, and a sense of job satisfaction all have an impact on their lives. Consequently, nurses are more susceptible to PMS [9]. Being present at work while sick is referred to as "sickness presenteeism," which can affect health and work productivity [11]. Sickness presenteeism is becoming a worldwide occupational health challenge in all occupations [11]. The severity of PMS was found to have a strong relationship with presenteeism [5].

The purpose of this study is to evaluate the impact of PMS on work-related quality of life among Jordanian nurses. By analyzing the effects of PMS on nurses' quality of life, this study hopes to open up conversations about how best to manage PMS in the workplace. This could potentially lead to better working conditions, improved work-life balance, and greater recognition of the physical and mental strain that PMS can cause.

Despite the critical importance of nurses’ work and the impact their quality of life has on their patients, very few studies on PMS and work-related quality of life among nurses have been conducted. This study could contribute to a better understanding of PMS symptoms and highlight the negative impact of PMS on the quality of life. It may help raise nurses’ awareness of the importance of treating symptoms and thus avoiding negative consequences. It may influence policy decisions, bringing the working environment in health facilities more in line with the health needs of female nurses.

## Materials and methods

Design, sample, and settings

Data were collected using a cross-sectional design from a convenience sample of 210 nurses working in the Jordan Ministry of Health hospitals (Princess Basmah Teaching Hospital, Princess Bade'a Teaching Hospital, and Princess Rahma Teaching Hospital). Female nurses between the ages of 18 and 45 years were our inclusion criteria. A diagnosis of depression or another mental illness, a diagnosis of a gynecological disease such as endometriosis, or pregnancy were all exclusion criteria.

Instruments

The data were collected from participants using a self-reported questionnaire (see Appendix) on Google Forms. It consists of the following three sections.

Demographic Factors and Menstrual Characteristics

This section included the sociodemographic and menstrual characteristics of the participants. Lifestyle factors were included and developed in accordance with existing literature [12], with some modifications tailored to the culture of the selected population.

Premenstrual Symptoms Screening Tool

The premenstrual symptoms screening tool (PSST) was used, which reflects and "converts" categorical Diagnostic and Statistical Manual of Mental Disorders, Fourth Edition (DSM-IV) standards into a ranking scale with levels of intensity [13]. The instrument consists of 14 PMS symptoms with a rank of four levels: not at all, mild, moderate, or severe. In addition, five other items were added regarding interference in daily life activities due to symptoms (work efficiency or productivity, relationships with co-workers, relationships with family, social life activities, and/or home responsibilities). The following standards should be found for a diagnosis of PMS [13]: no less than one of the first four symptoms should be moderate to severe, no less than four symptoms must be moderate to severe, and no less than one of the standards about daily life function impairment should be moderate to severe.

The reliability of the tool in our study was tested, and Cronbach’s alpha coefficient was 0.95.

Work-Related Quality of Life Scale

This tool was used to measure the work-related quality of life among Jordanian nurses. It includes 24 items on a 5-point Likert scale (from 1 = "strongly agree" to 5 = "strongly disagree") and six subscales: General Well-Being (GWB), Home-Work Interface (HWI), Job Career Satisfaction (JCS), Control at Work (CAW), Working Conditions (WCS), and Stress at Work (SAW). Each subscale score is determined by calculating the average of the item scores contributing to that factor, with the scores reversed for the three negatively phrased items (questions 7, 9, and 19). The 24th item is not used in the calculation as it is used to evaluate the reliability and validity of the scale. The overall Work-Related Quality of Life Scale (WRQoL) score is determined by calculating the sum of the six subscale scores; as the scores increase, the work-related quality of life also increases [14]. The reliability of the tool in our study was tested, and Cronbach’s alpha coefficient for the overall scale was 0.91.

To ensure accuracy and validity, all questionnaire sections have been translated into Arabic and validated by professionals in public health.

Informed consent and ethical approval

A consent form was obtained from each nurse who met the eligibility criteria. The informed consent has information explaining the study purpose, procedure, and participant’s rights. The nurses were assured that their participation was voluntary and that they could withdraw at any time without any explanation. Also, the nurses were assured that their responses would be kept confidential and not shared with anyone, and only the researcher could access the data. Ethical approval was obtained from the Jordan Ministry of Health (approval number: 18687).

Statistical analysis

Statistical Package for Social Sciences (SPSS) Version 25 (IBM Corp., Armonk, NY) was used for data entry, cleaning, and analysis. The mean and standard deviation were used to describe the continuous variables, and frequencies and percentages were used for the categorical variables. Participants were divided into two groups according to the presence or absence of PMS, and then demographics, menstrual history, and WRQoL scores were compared using the chi-square test and t-tests. Multivariable logistic regression was used to assess the potential relationship between the study variables and the PMS variable. A p-value of 0.05 or less was considered statistically significant. Adjusted odds ratios (AORs) and 95% confidence intervals (95% CIs) were presented.

## Results

This study had 210 participants. Their mean age was 34.88 years (SD = 5.20), they were mostly married (86.2%), and 65.2% lived in a city. In terms of job specifications, the majority of participants (n = 118, 56.2%) were registered nurses with bachelor's degrees (n = 115, 54.8%), worked 41.07 (SD = 4.94) hours per week on average, and 59% worked night shifts. Also, 11.4% used combined oral contraceptive pills (COCP), 7.6% smoked cigarettes, and 18.6% smoked waterpipes. Table 1 contains a detailed description of the participants' characteristics. Anger and irritability were the most commonly reported effective symptoms of PMS, followed by somatic symptoms such as muscle and joint pain. It demonstrated that PMS had a significant impact on nurses' family relationships and social activities.

**Table 1:** Distribution of study participants by background characteristics (N = 210) COCP, combined oral contraceptive pills; DM, diabetes mellitus; HTN, hypertension; SD, standard deviation; BMI, body mass index

Demographic variables		Numbers (n)	Percentage (%)
Marital status	Never married	29	13.8
	Ever married	181	86.2
Number of children	No children	36	17.1
	One child	27	12.9
	Two children	39	18.6
	Three children	42	20
	More than three children	66	31.4
Residence	Rural	73	34.8
	Urban	137	65.2
Job title	Registered nurse	118	56.2
	Others	92	43.8
Educational level	Diploma	68	32.4
	Bachelor	115	54.8
	Postgraduate	27	12.9
Years of experience	Less than 5 years	47	22.4
	5-10 years	66	31.4
	More than 10 years	97	46.2
Night shifts	Yes	124	59
	No	86	41
Income satisfaction	Dissatisfied	129	61.4
	Satisfied	81	38.6
Coffee intake	Yes	134	63.8
	No	76	36.2
Use of COCP	Yes	24	11.4
	No	186	88.6
Chronic diseases	Yes (DM, HTN, thyroid)	45	21.4
	No	165	78.6
Smoking	Yes	52	24.8
	No	158	75.2
Cigarette smoking	Yes	16	7.6
	No	194	92.4
Waterpipe smoking	Yes	39	18.6
	No	171	81.4
Age, mean (SD)	34.88 (5.20)		
Working hours, mean (SD)	41.07 (4.94)		
BMI, mean (SD)	25.87 (4.38)		

The majority of participants (61%) began menstruating between 12 and 14 years of age with a regular cycle (77.1%) and a medium flow (n = 144, 68.6%). Almost half (47.6%) reported a family history of PMS. As shown in Table 2, 60.5% of women had experienced PMS. The overall WRQoL mean score was 67.47 (SD = 15.20). This study discovered a significant association of PMS with age, night shift work, COCP use, smoking, dysmenorrhea, and family history, as shown in Tables 3-5.

**Table 2:** Distribution of study participants by menstrual characteristics and treatment methods (N = 210) PMS, premenstrual syndrome

Menstrual characteristics variables		Numbers (n)	Percentage %
Age at menarche	Less than 11 years	14	6.7
	12-14 years	128	61
	More than 15 years	68	32.4
Cycle regularity	Yes	162	77.1
	No	48	22.9
Length of menstruation	Less than 3 days	12	5.7
	3-8 days	175	83.3
	More than 8 days	23	11
Length of cycle	Less than 21 days	20	9.5
	21-35 day	167	79.5
	More than 35 days	23	11
Amount of flow	Scanty	10	4.8
	Medium	144	68.6
	Heavy	56	26.7
Dysmenorrhea	Absent	20	9.5
	Mild pain	56	26.7
	Moderate pain	75	35.7
	Severe pain	59	28.1
Family history of PMS	Yes	100	47.6
	No	110	52.4

**Table 3:** Distribution of participants by PMS and background characteristics (N=210) The chi-square test was used. *Statistical significance was achieved at p < 0.05. COCP, combined oral contraceptive pills; DM, diabetes mellitus; HTN, hypertension; PMS, premenstrual syndrome

Demographic factor	PMS (no), n (%)	PMS (yes), n (%)	Total, n (%)	p-Value
Marital status				
Never married	6 (20.7)	23 (79.3)	29 (100)	0.025*
Ever married	77 (42.5)	104 (57.5)	181 (100)	
No. of children				
None	8 (22.2)	28 (77.8)	36 (100)	0.031*
One child	9 (33.3)	18 (66.7)	27 (100)	
Two children	13 (33.3)	26 (66.7)	39 (100)	
Three children	23 (54.8)	19 (45.2)	42 (100)	
More than three children	30 (45.5)	36 (54.5)	66 (100)	
Residence				
Rural	36 (49.3)	37 (50.7)	73 (100)	0.034*
Urban	47 (34.3)	90 (65.7)	137 (100)	
Job title				
Registered nurse	49 (41.5)	69 (58.5)	118 (100)	0.502
Others	34 (37)	58 (63)	92 (100)	
Educational level				
Diploma	28 (41.2)	40 (58.8)	68 (100)	0.301
Bachelor	48 (41.7)	67 (58.3)	115 (100)	
Postgraduate	7 (25.9)	20 (74.1)	27 (100)	
Years of experience				
Less than 5 years	16 (34.0)	31 (66.0)	47 (100)	0.000*
5-10 years	15 (22.7)	51 (77.3)	66 (100)	
More than 10 years	52 (53.6)	45 (46.4)	97 (100)	
Night shifts				
Yes	41 (33.1)	83 (66.9)	124 (100)	0.022*
No	42 (48.8)	44 (51.2)	86 (100)	
Income satisfaction				
Dissatisfied	49 (38.0)	80 (62.0)	129 (100)	0.565
Satisfied	34 (42.0)	47 (58.0)	81 (100)	
Coffee intake				
Yes	47 (35.1)	87 (64.9)	134 (100)	0.08
No	36 (47.4)	40 (52.6)	76 (100)	
Use of COCP				
Yes	4 (16.7)	20 (83.3)	24 (100)	0.015*
No	79 (42.5)	107 (57.5)	186 (100)	
Chronic diseases				
Yes (DM, HTN, thyroid)	16 (35.6)	29 (64.4)	45 (100)	0.539
No	67 (40.6)	98 (59.4)	165 (100)	
Smoking				
Yes	11 (21.2)	30 (57.7)	52 (100)	0.002*
No	72 (45.6)	86 (54.4)	158 (100)	
Cigarette smoking				
Yes	4 (25)	12 (75)	16 (100)	0.216
No	79 (40.7)	115 (59.3)	194 (100)	
Waterpipe				
Yes	8 (20.5)	31 (79.5)	39 (100)	0.007*
No	75 (43.9)	96 (56.1)	171 (100)	

**Table 4:** Distribution of participants by PMS and continuous demographic variables (N=210) The independent sample t-test was used. *Statistical significance was achieved at p < 0.05. PMS, premenstrual syndrome; SD, standard deviation

Demographic factor	PMS (no)		PMS (yes)		p-Value
	Mean	SD	Mean	SD	
Age	36.6	4.55	33.76	5.3	0.000 *
No. of working hours	40.88	4.1	41.2	4.92	0.65
Body mass index	26.79	3.99	25.27	4.53	0.013 *

**Table 5:** Distribution of participants by PMS status and by menstrual characteristics and treatment methods (N=210) the chi-square test was used. *Statistical significance was achieved at p < 0.05. PMS, premenstrual syndrome

Menstruation data	PMS (no), n (%)	PMS (yes), n (%)	Total, n (%)	p-Value
Age of menarche				
≤11 years	6 (42.9)	8 (57.1)	14 (100)	0.841
12-14 years	52 (40.6)	76 (59.4)	128 (100)	
≤15 years	25 (36.8)	43 (63.2)	68 (100)	
Cycle regularity				
Yes	66 (40.7)	96 (59.3)	162 (100)	0.508
No	17 (35.4)	31 (64.6)	48 (100)	
Length of menstruation				
Less than 3 days	5 (41.7)	7 (58.30)	12 (100)	0.181
3-8 days	73 (41.7)	102 (58.30)	175 (100)	
More than 8 days	5 (21.7)	18 (78.30)	23 (100)	
Length of cycle				
Less than 21 days	5 (25)	15 (75)	20 (100)	0.293
21-35 day	67 (40.1)	100 (59.9)	167 (100)	
More than 35 days	11 (47.8)	12(52.2)	23 (100)	
Amount of flow				
Scanty	6 (60)	4 (40)	10 (100)	0.001*
Medium	66 (45.8)	78 (54.2)	144 (100)	
Heavy	11 (19.6)	45 (80.4)	56 (100)	
Dysmenorrhea				
Absent	12 (60)	8 (40)	20 (100)	0.000*
Mild pain	33 (58.9)	23 (41.1)	56 (100)	
Moderate pain	34 (45.3)	41 (54.7)	75 (100)	
Severe pain	4 (6.8)	55 (93.2)	59 (100)	
Family history of PMS				
Yes	22 (22)	78 (78)	100 (100)	0.000*
No	61 (55.5)	49 (44.5)	110 (100)	

The WRQoL scores of participants differed statistically in four of the six WRQoL subscales: job and career satisfaction, control at work, home-work interface, and stress at work. There was a significant difference in mean total WRQoL score between the two groups, with nurses with PMS having a lower mean total WRQoL score (mean = 65.47, SD = 15.38) than nurses without PMS (mean = 70.54, SD = 14.47), as shown in Table 6.

**Table 6:** Distribution of participants by PMS and WRQoL (N=210) The independent sample t-test was used. *Statistical significance was achieved at p < 0.05. PMS, premenstrual syndrome; SD, standard deviation; WRQoL, Work-Related Quality of Life scale

Subscales	PMS (no)		PMS (yes)		P-Value
Continuous variables	Mean	SD	Mean	SD	
Job and career satisfaction	19.61	4.76	17.31	4.75	0.001^*^
Control at work	9.57	2.81	8.63	2.89	0.021^*^
General well-being	18.60	3.57	17.60	4.04	0.067
Home-work interface	8.53	2.77	7.57	2.53	0.011^*^
Stress at work	5.59	2.04	6.43	2.05	0.004^*^
Working conditions	8.64	2.97	7.92	2.66	0.070
WRQoL score	70.54	14.47	65.47	15.38	0.018^*^

Using the backward selection method, the regression model identified job and career satisfaction and workplace stress as significant predictors of PMS after controlling for the effects of other variables (age, use of COCP, family history, and dysmenorrhea). Those who were more satisfied with their jobs and careers were less likely to have PMS (AOR = 0.92, p = 0.026, 95% CI = 0.85, 0.99), while those who were more stressed at work were more likely to have PMS (AOR = 1.2, p = 0.036, 95% CI = 1.01, 1.42). After controlling for the effect of other variables in the model, the model revealed that one year of age increase reduces the risk of PMS by 0.90 (p = 0.002, 95% CI = 0.84, 0.96).

Moreover, nurses who used COCP were 5.18 (p = 0.018, 95% CI = 1.33, 20.17) times more likely to have PMS than nurses who did not. Moreover, having a first-degree relative with PMS increases the risk of PMS by 2.52 times (p = 0.012, 95% CI = 1.23, 5.18). In addition, severe dysmenorrhea was associated with an 11.78-fold increase in the risk of PMS (p = 0.002, 95% CI = 2.48, 56.02), as shown in Table 7.

**Table 7:** Regression model of PMS and variables *Statistical significance was achieved at p < 0.05. AOR, adjusted odds ratio; CI, confidence interval; COCP, combined oral contraceptive pills

Variables	AOR	95% CI		p-Value
Age	0.90	0.84	0.96	0.002^*^
Use of COCP	5.18	1.33	20.17	0.018^*^
Family history	2.52	1.23	5.18	0.012^*^
Dysmenorrhea (absent)	-	-	-	0.002^*^
Mild pain	1.24	0.35	4.33	0.741
Moderate pain	1.52	0.43	5.34	0.512
Severe pain	11.78	2.48	56.02	0.002^*^
Job and career satisfaction	0.92	0.85	0.99	0.026
Stress at work	1.20	1.01	1.42	0.036^*^
Constant	35.29	-	-	0.033

## Discussion

In this study, 60.5% of nurses reported having PMS. A study of Turkish nurses reported that 38.1% of them suffered from PMS [9]. PMS prevalence is associated with a variety of factors, including sample size, participant characteristics, and differences in culture. Differences in diagnostic tools, as well as its diagnoses, have quite a broad range of definitions and cut points. [9,15].

There was no significant relationship between PMS and job title in terms of job history. This is because all staff, including registered nurses, midwives, assistants, and practical nurses, are assigned an equal workload. The educational level of the participants was unimportant because they were all educated in nursing colleges at various levels and were assumed to be aware of the PMS condition. A study of working women in Thailand, on the other hand, found that those with higher education had more PMS than those with lower education. In contrast to our study, half of the participants had only completed primary or secondary school [16].

Furthermore, experience duration was found to be significantly related to PMS, with a high prevalence in those with less than 10 years of experience. This finding is consistent with the age results, which show that PMS is more prevalent in younger nurses. Additionally, working hours had no effect on the presence of PMS, whereas night shifts did. These findings show that despite working the same number of hours each week, those who worked night shifts had more PMS. These findings are consistent with the findings of a study that investigated the relationship between sleep and PMS and discovered that women with PMS have a higher rate of poor-quality sleep [17]. Furthermore, a Korean study found that sleep time variability and poor sleep quality exacerbated nurses' PMS symptoms [18]. Nurses' sleep quality deteriorates as a result of working night shifts, increasing the risk of PMS.

PMS detrimentally affects an individual's quality of life. It has been shown to have an impact on all aspects of a woman's life, including daily activity, social life, study, and work productivity [3,8,19]. Our findings back up what others have discovered. In nurses suffering from PMS, the WRQoL score was significantly lower. PMS has a significant impact on working women [5]. Furthermore, because of their difficult working conditions, female nurses are more susceptible to PMS [9].

Because it is an indicator of job satisfaction, nurses' work ability is an important aspect of the quality of their work life. Work ability has been shown to be significantly related to WRQoL score, implying that tracking WRQoL and nurses' work ability would assist hospitals in determining their status and taking steps to improve working conditions [20]. As a result, nurses suffering from PMS experience a wide range of symptoms that can impair their ability to work.

In our study, we discovered that working the night shift increases the risk of developing PMS in nurses, which can lead to a decrease in WRQoL. A study found that nurses who did not work night shifts had a significantly higher quality of work life [21]; thus, night shifts, PMS, and WRQoL form a vicious circle. New regulations are required to improve the quality of life for female nurses at work. Reducing the number of night shifts, for example, can help female nurses with PMS have a better working experience.

Control at work and stress at work were found to be significantly associated with PMS, and stress at work was found to be a predictor of it in the logistic regression. PMS is a sensitive subject for women. According to one study, it is difficult to disclose the reason for needing rest due to PMS symptoms, and many managers did not believe that PMS symptoms were a valid reason for taking time off from work [5]. Furthermore, having a male manager may heighten the embarrassment of reporting PMS as a reason for sick leave. Even if the manager was female but did not have PMS, nurses may feel unsupported [5]. All of these factors can lead to nurses feeling out of control at work and increasing their stress levels, causing PMS symptoms to exacerbate the problem.

Furthermore, nurses reported that PMS has an impact on their relationships with their families as well as their social activities. This is consistent with a significant reduction in the home-work interface scale in PMS nurses. When nurses fail to balance work and home responsibilities, their ability to get the most out of either domain is jeopardized. Job and career satisfaction were also significantly associated with PMS. Logistic regression discovered an inverse relationship between them. It is possible to conclude that hospitals' inability to achieve job satisfaction for female nurses with PMS is due to their having to work long hours, meeting unforeseen work demands, a lack of manpower, and, more specifically, their job dissatisfaction [14].

Our findings should be considered by healthcare executives who are responsible for improving their employees' work-related quality of life. Female nurses may be subject to additional regulations. Measures are needed, for example, to reduce sleep time variability and improve sleep quality, which can reduce workplace stress. Listening to nurses to determine their needs and involving them in decision-making can also increase a sense of control at work. Reducing restrictions on female nurses' sick leave and staff shortages can enhance nurses' satisfaction. To achieve work-life balance and focus on increasing that balance qualitatively, colleagues and managers must consider each nurse's life circumstances individually and create a supportive environment in which nurses can strive for a better work-life balance. This would allow nurses to sustain their desired level of productivity while also increasing their satisfaction at work and at home.

According to the current study, PMS is quite common among Jordanian nurses, with a negative impact on their work-related quality of life. The most commonly reported effective PMS symptoms were anger and irritability, followed by muscle and joint pain. It was discovered that PMS had a significant impact on nurses' family relationships and social activities. Furthermore, various demographic and menstrual characteristics were discovered to be significantly associated with PMS. Logistic regression models discovered that increasing age, family history, using COCP, and having severe dysmenorrhea are all risk factors for PMS. Furthermore, it was discovered that workplace stress has a direct relationship with PMS, whereas job and career satisfaction have an inverse relationship with PMS.

Limitations

There are several limitations of this study. We are cognizant of the possibility of self-report bias since the information used in this study was based on participant self-reports of their symptoms (under- or over-reporting of PMS symptoms). The results also cannot be applied to all industries because different work circumstances may exist that interfere with work-related quality of life because the study was only conducted in the public sector.

## Conclusions

Overall, nurses with PMS are dissatisfied with their career opportunities, face excessive pressure at work, experience increased stress at work, have a work-life balance, are less involved in decisions that affect their lives, and have little sense of control at work. As a result, the work-related quality of life decreases. We recommend increasing nurses' awareness of the disease, its symptoms, and effects on WRQoL. In order to take appropriate action, the study should be widened to encompass all female health care workers in both the public and private sectors to identify the differences. Policymakers should also be educated about the disease in order to persuade them to consider enacting or passing new legislation.

## Appendices

Part 1    Demographic and menstrual characteristics

Instructions: Please read the following carefully and answer them by placing () mark in the bracket against the response relevant to you and fill in the blanks.

1.     Age ….…

2.     Marital status:

a.     Married

b.     Single

c.     Divorced

d.     Widowed

3.     How many children do you have?

a.     One child

b.     Two children

c.     Three children

d.     More than three children

e.     I don’t have any children

4.     Residence:

a.      Rural area

b.     Urban area

5.     Occupation:

a.      Staff nurse

b.     Practical nurse

c.      Assistant nurse

d.     Midwife

e.      Executive staff

6.     Educational level:

a.      College

b.     Bachelor

c.      Higher studies (master’s degree, doctoral degree)

7.     Number of working years:

a.      <5 years

b.     5-10 years

c.      >10 years

8.     Number of working hours/weeks ….…

9.     Does your work include night shift?

a.      Yes

b.     No

10.    Are you satisfied with the income?

a.      Very satisfied

b.     Satisfied

c.      Neutral

d.     Dissatisfied

e.      Very dissatisfied

11.     Weight in kilograms (kg) ….…

12.     Height (in centimeters) ….…

13.     Age at menarche (in years) ….…

14.     Is your period regular?

a.      Yes

b.     No

15.     How many days does your menstrual cycle last?

a.      <3 days

b.     3-8 days

c.      >8 days

16.     What is the length of your menstrual cycle?

a.      <21 days

b.     21-35 days

c.      >35 days

17.     How do you evaluate the amount of flow?

a.      Heavy

b.     Medium

c.      Scanty

18.     Do you have a complaint of dysmenorrhea?

a.      Absent

b.     Mild pain

c.      Moderate pain

d.     Severe pain

19.     How do you relief the symptoms of menstrual cycle?

a.       Pharmacological methods (analgesic medications)

b.       Non-pharmacological methods (home remedies, warm water compresses)

c.      Both

d.      Didn’t use any methods

20.    Which non-pharmacological methods do you use?

a.      Home remedies

b.     Warm water compresses

c.      Taking rest

d.     Not using non-pharmacological methods

e.      Others ….…

21.     Do you smoke?

a.      Waterpipe

b.     Cigarette smoking

c.      Non-smoker

22.     Do you drink coffee regularly?

a.      Yes

b.     No

23.     Do you take COCP (combined oral contraceptive pills)?

a.      Yes

b.     No

24.     Have you been diagnosed with a chronic illness?

a.      No

b.     Hypertension

c.      Diabetes mellitus

d.     Thyroid diseases

e.      Others ….…  

25.     Do your first-degree relatives (mother, sister) complain of PMS?

a.      Yes

b.     No

Part 2: Premenstrual symptoms screening tool (PSST) and physical symptoms

Instructions: Answer the following questions. Mark the circle against the response relevant to you from the following options.

Do you experience some or any of the following premenstrual symptoms which start before your period and stop within a few days of bleeding?

**Table 8:** Premenstrual symptoms screening tool: part 1

Symptoms	Not at all	Mild	Moderate	Severe
1. Anger/irritability				
2. Anxiety/tension				
3.Tearful/increased sensitivity to rejection				
4. Depressed mood/hopelessness				
5. Decreased interest in work activities				
6. Decreased interest in home activities				
7. Decreased interest in social activities				
8. Difficulty concentration				
9. Fatigue/lack of energy				
10. Overeating/food cravings				
11. Insomnia				
12. Hypersomnia (needing more sleep)				
13. Feeling overwhelmed or out of control				
14. Physical symptoms				

**Table 9:** Premenstrual symptoms screening tool: part 2

Have your symptoms, as listed above, interfered with:	Not at all	Mild	Moderate	Severe
A. Your work efficiency or productivity				
B. Your relationships with coworkers				
C. Your relationships with your family				
D. Your social life activities				
E. Your home responsibilities				

Part 3: Work-Related Quality of Life (WRQoL) Scale

Instructions: Answer the following questions. Mark the circle against the response relevant to you from the following options.

Please do not take too long over each question; we want your first reaction not a long drawn-out thought process. Please do not omit any questions. This isn’t a test, simply a measure of your attitudes to the factors that influence your experience at work.

**Table 10:** Work-Related Quality of Life (WRQoL) Scale

To what extent do you agree with the following?	Strongly disagree	Disagree	Neutral	Agree	Strongly agree
1. I have a clear set of goals and aims to enable me to do my job					
2. I feel able to voice opinions and influence changes in my area of work					
3. I have the opportunity to use my abilities at work					
4. I feel well at the moment					
5. My employer provides adequate facilities and flexibility for me to fit work in around my family life					
6. My current working hours/patterns suit my personal circumstances					
7. I often feel under pressure at work					
8. When I have done a good job, it is acknowledged by my line manager					
9. Recently, I have been feeling unhappy and depressed					
10. I am satisfied with my life					
11. I am encouraged to develop new skills					
12. I am involved in decisions that affect me in my own area of work					
13. My employer provides me with what I need to do my job effectively					
14. My line manager actively promotes flexible working hours/patterns					
15. In most ways my life is close to ideal					
16. I work in a safe environment					
17. Generally things work out well for me					
18. I am satisfied with the career opportunities available for me here					
19. I often feel excessive levels of stress at work					
20. I am satisfied with the training I receive in order to perform my present job					
21. Recently, I have been feeling reasonably happy all things considered					
22. The working conditions are satisfactory					
23.I am involved in decisions that affect members of the public in my own area of work					
24. I am satisfied with the overall quality of my working life					
